# Circulating Tumor Cells in Desmoid Tumors: New Perspectives

**DOI:** 10.3389/fonc.2021.622626

**Published:** 2021-09-14

**Authors:** Alexcia C. Braun, Fernando A. B. Campos, Emne A. Abdallah, Anna P. C. Ruano, Tiago da S. Medina, Milena S. Tariki, Fabio F. E. Pinto, Celso A. L. de Mello, Ludmilla T. D. Chinen

**Affiliations:** ^1^International Center for Research, A.C. Camargo Cancer Center, São Paulo, Brazil; ^2^Department of Clinical Oncology, A. C. Camargo Cancer Center, São Paulo, Brazil; ^3^Department of Orthopedics, A. C. Camargo Cancer Center, São Paulo, Brazil

**Keywords:** circulating tumor cells, desmoid tumor, beta catenin expression, vimentin expression, TGF-βRI expression

## Abstract

**Introduction:**

Desmoid tumor (DT) is a rare neoplasm with high local recurrence rates, composed of fibroblastic cells that are characterized by the expression of key molecules, including the intermediate filament vimentin, cyclooxygenase-2 (COX-2), and nuclear β-catenin, and lack of epithelial markers. Circulating tumor cells (CTCs) isolated from the peripheral blood of patients with sarcomas and other neoplasms can be used as early biomarkers of tumor invasion and dissemination. Moreover, CTCs can also re-colonize their tumors of origin through a process of “tumor self-seeding.”

**Objectives:**

We aimed to identify CTCs in the peripheral blood of patients with DT and evaluate their expression of β-catenin, transforming growth factor receptor I (TGF-βRI), COX-2, and vimentin proteins.

**Material and Methods:**

We conducted a prospective study of patients with initial diagnosis or relapsed DT with measurable disease. Blood samples from each patient were processed and filtered by ISET^®^ (Rarecells, France) for CTC isolation and quantification. The CTC expression of β-catenin, COX-2, TGF-βRI, and vimentin was analyzed by immunocytochemistry (ICC).

**Results:**

A total of 18 patients were included, and all had detectable CTCs. We found a concordance of β-catenin expression in both CTCs and primary tumors in 42.8% (6/14) of cases by using ICC and immunohistochemistry, respectively.

**Conclusions:**

Our study identified a high prevalence of CTCs in DT patients. Concordance of β-catenin expression between primary tumor and CTCs brings new perspectives to assess the dynamics of CTCs in the blood compartment, opening new avenues for studying the biology and behavior of DT. In addition, these results open the possibility of using CTCs to predict DT dynamics at the time of disease progression and treatment. Further studies with larger sample sizes are needed to validate our findings.

## Introduction

Desmoid tumor (DT) is a rare non-metastasizing mesenchymal neoplasm that can show aggressive local behavior and thereby can impact the functionality and quality of life of the patients (1, 2). DTs are typically diagnosed in adults (35–40 years) and are more common in women of reproductive age (3, 4). They can affect any anatomic site but are commonly localized in the extremities, abdominal wall, and abdominal mesentery (5). The clinical outcomes of DTs are unpredictable, with some progressing to large-sized tumors with infiltration and destruction of adjacent vital structures, and others showing spontaneous regression (6). Nevertheless, reliable and validated predictive factors regarding DT evolution are still lacking (7).

The initial management of asymptomatic patients with non-life-threatening DTs is often watch-and-wait, as up to 20% will regress spontaneously (8). If treatment is indicated, options include surgery, radiation, or systemic therapy with tyrosine-kinase inhibitors or chemotherapy (6, 8). Due to the infiltrative nature of the disease, post-surgical recurrence rates can exceed 50% of cases, especially for extra-abdominal desmoids, which reinforce observation as an adequate strategy (9).

Histologically, DTs are composed of fibroblastic cells characterized on immunohistochemistry (IHC) by the expression of intermediate filament vimentin, along with cyclooxygenase-2 (COX-2) and nuclear β-catenin, and lack of epithelial markers expression (1, 10, 11). Approximately 90% of DTs are sporadic, while the remaining cases are usually related to familial adenomatous polyposis (FAP), specifically Gardner syndrome (12). In both cases, mutations that activate the Wnt/β-catenin signaling pathway are likely to play a significant role in tumorigenesis (13). Wnt/β-catenin signaling is involved in numerous processes, such as the control of gene expression and regulation of cell adhesion and polarity (14). In addition, approximately 85% of DTs are related to mutations in the exon 3 of the beta-catenin encoding gene *CTNNB1* (15). *CTNNB1* mutations of DTs generally occur at codons 41 or 45, with p.T41A (threonine to alanine), p.S45F (serine to phenylalanine), and p.S45P (serine to proline) being the most frequent ones (15–21).

Used as a new tool for diagnosing and monitoring cancer, liquid biopsies have received increasing attention. Using a simple blood test has enormous implications for the diagnosis of cancer, so it is possible to avoid invasive tissue biopsies in the future and obtain a similar result from the circulating tumor cell (CTC) test (22).

CTCs play a central role on tumor dissemination and metastasis, which are ultimately responsible for most cancer deaths. Cancer cells can enter circulation years before a tumor is diagnosed. The majority of cells die, and only a minor fraction contains viable metastatic precursors that infiltrate organs and survive for eventual relapse (23–25). The presence of CTCs and/or circulating tumor microemboli (CTMs) in the peripheral blood of patients can be early markers of tumor invasion and spread (26). The dissemination of cancer cells from a primary tumor is conventionally viewed as a unidirectional process that culminates with the metastatic colonization of distant organs. CTCs and CTMs are associated with poor prognosis in several carcinomas. Furthermore, CTCs can colonize their tumors of origin through a process of “tumor self-seeding” (27). In contrast to carcinomas, few studies have examined CTCs and CTMs in mesenchymal tumor patients (28), and to the best of our knowledge, there are no reports of CTC detection in cases of DT.

Therefore, the aims of this study were to determine whether CTCs could be detected in DT patients and analyze their expression of mesenchymal proteins [vimentin, transforming growth factor receptor I (TGF-βRI)], as well as β-catenin and COX-2.

## Methods

### Patients and Samples

This was a prospective and descriptive study, conducted with DT patients treated at the A.C. Camargo Cancer Center, São Paulo, Brazil, between June 2017 and October 2019. Peripheral blood samples were obtained after written informed consent. CTCs were analyzed by ISET^®^ assay (Rarecells Diagnostics, Paris, France). This study was approved by the local Research Ethics Board (REB protocol 2427/17). Inclusion criteria were age >18 years, diagnosis of DT, presence of measurable disease, and a negative medical history of recent surgical procedures or trauma. Patients with FAP were excluded.

### ISET^®^ Assay

Blood samples (8 ml) were drawn in EDTA tubes (BD Vacutainer^®^) with immediate gentle agitation after blood collection. If samples were not processed immediately after phlebotomy, the tubes were left on a blood homogenizer at room temperature for up to 4 h. The isolation by size of epithelial tumor cells (ISET) assay was performed as described previously (29). The samples were processed on the platform according to the manufacturer’s instructions. Eight milliliter of whole blood (1 ml per spot) was topped up to 80 ml with erythrocyte lysis buffer containing 0.02% formaldehyde, incubated for 10 min at room temperature, and filtered through an 8-µm pore membrane. To preserve cell integrity, filtration pressure was optimized at −10 kPa. The membrane was then washed once with phosphate-buffered saline 1×. After processing, membranes were dried, wrapped in aluminum foil, and stored at −20°C until use.

### Immunocytochemistry

The spot membranes were subjected to immunocytochemistry (ICC) in 24-well plates to evaluate protein expression. We made single [one protein analyzed, stained with DAB (Dako™)] or double (two proteins analyzed, stained with DAB and magenta chromogen) ICC assays. Before ICC reactions, antigen retrieval was performed using Antigen Retrieval Solution (Dako™). Cells in the ISET spots were then hydrated with tris-buffered saline (TBS) 1× for 20 min and permeabilized with TBS + Triton X-100 for 5 min. Endogenous peroxides were blocked with 3% hydrogen peroxide and kept in the dark for 15 min. The spots were incubated with antibodies diluted in TBS supplemented with 10% fetal calf serum. For single staining, antibody staining was visualized with DAB (Dako™). To amplify the antibody signal for double staining, the spots were incubated with Envision G/2 Doublestain System, Rabbit/Mouse (Dako™), followed by a 10-min incubation with magenta chromogen to visualize the second antibody. Cells were stained with hematoxylin and analyzed by light microscopy (BX61-Olympus).

Negative and positive controls were added for each ICC staining. For positive controls, blood from healthy subjects was spiked with A549 and U-87 cell lines. According to the Human Protein Atlas (http://www.proteinatlas.org/), the A549 cell line expresses both β-catenin and TGF-βRI, whereas the U-87 cell line expresses both COX-2 and vimentin. These cell lines were acquired from ATCC^®^ HTB-43™. For negative controls, A549 and U-87 cell lines spiked in healthy blood were not incubated with the primary antibody, to avoid cross-reactivity (**Supplementary Figure 1**). For protein expression analysis, cells were classified according to their staining characteristics. The absence of staining was considered negative, while staining of the nucleus or cytoplasm was classified as positive, as expected for each antibody. We did not perform statistical analysis to evaluate protein expression, progression-free survival, or overall survival due to the rarity of events in these patients and the short follow-up interval.

## Results

### Detection of CTCs and Protein Expression Analysis

Recently, it was shown that the limit of detection of ISET is 1 CTC/10 ml of whole blood and that despite its high sensitivity, the distribution of CTCs in membrane spots is not uniform (30). Thus, we tested all eight spots fixed with 0.02% formaldehyde to ensure the observation of all isolated CTCs.

Eighteen patients were enrolled in this study, including two males and 16 females, with median age at the time of recruitment of 43.5 years (18–65 years). DTs were diagnosed in the lower extremities of 27.7% of patients. Clinical and pathological characteristics were obtained from medical records and are summarized in **Table 1**.

**Table 1:** Clinical characteristics of the patients.

Variable	No.	%
**Total number of patients**	18	100
**Age at recruitment, years**		
Median (range)	43.5 (18–65)	
**Gender**		
Male	2	11
Female	16	89
**Tumor location**		
Lower limbs	5	28
Upper limbs	2	11
Supraclavicular	2	11
Abdominal wall	3	18
Scapular region	1	5
Retroperitoneum	1	5
Gluteal region	2	11
Paravertebral	2	11
**Relapse**		
Yes	12	67
No	6	33

**Figure 1:**
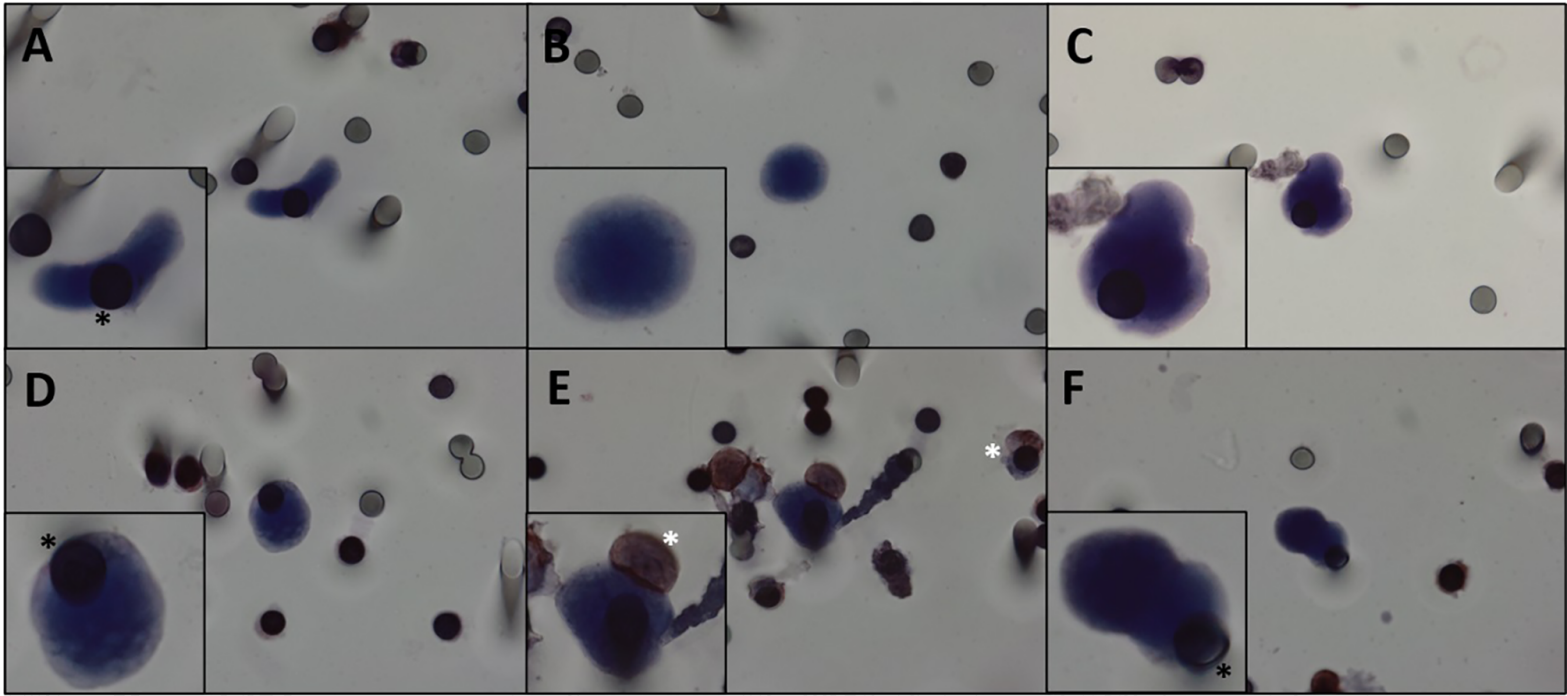
**(A–F)** CTCs isolated from patients with DTs. Cells visualized with hematoxylin. We can observe pleomorphic desmoid tumor CTCs with all cytopathological features: nucleus size ≥ 12 µm, hyperchromatic and irregular nucleus, visible presence of cytoplasm, and a high nucleus–cytoplasm ratio (31). White asterisk: leukocytes. Black asterisk: membrane spots. Images were taken at ×600 magnification using a light microscope (Research System Microscope BX61—Olympus, Tokyo, Japan) coupled to a digital camera (SC100—Olympus, Tokyo, Japan). CTCs, circulating tumor cells; DTs, desmoid tumors.

CTCs were detected in all patients. Eight spots were tested for each patient. Two spots were tested for β-catenin and vimentin expression (double staining), two were single-stained for either COX-2 or TGFβ-RI, and the last spots were stained with hematoxylin for CTC visualization. In addition, the estimated CTC count was made considering the average count of the eight spots evaluated. The average number of CTCs detected by ISET^®^ was 2.6 CTC/ml (0.5–13 CTCs/ml). One CTM, a cluster of three or more CTCs, was found in one patient. Quantification and characterization of CTCs are presented in **Table 2**, **Figure 1**, and **Supplementary Figure 2**.

**Table 2:** CTC counting and characterization.

Patient ID	β-Catenin expression in the primary tumor	Number of β-catenin-positive CTCs in 5 ml of blood	Number of TGFβ-RI-positive CTCs in 5 ml of blood	Number of vimentin-positive CTCs in 5 ml of blood	Number of COX-2-positive CTCs in 5 ml of blood	CTC/1 ml
1	Negative	1	0	0	1	1
2	Positive	4	0	0	1	1.8
3	Positive	0	2	2	0	3.8
4	Positive	0	1	0	2	1
5	Positive	4	0	2	2	2.6
6	Positive	0	0	0	0	0.8
7	Positive	0	4	0	8	13
8	Positive	2	1	0	0	4.8
9	Positive	0	0	0	0	2.6
10	Positive	5	8	1	0	5
11	-	2	7	2	0	4.75
12	Negative	0	0	0	0	0.5
13	-	4	2	0	0	1.75
14	Negative	1	3	0	0	10.5
15	-	3	0	0	1	5
16	–	1	0	0	0	1.25
17	Positive	6*	0	9*	1	8
18	Positive	0	1	0	0	1

CTC, circulating tumor cell; COX-2, cyclooxygenase-2.

*Patient with positive circulating tumor microemboli (CTMs) for β-catenin and vimentin.

**Figure 2:**
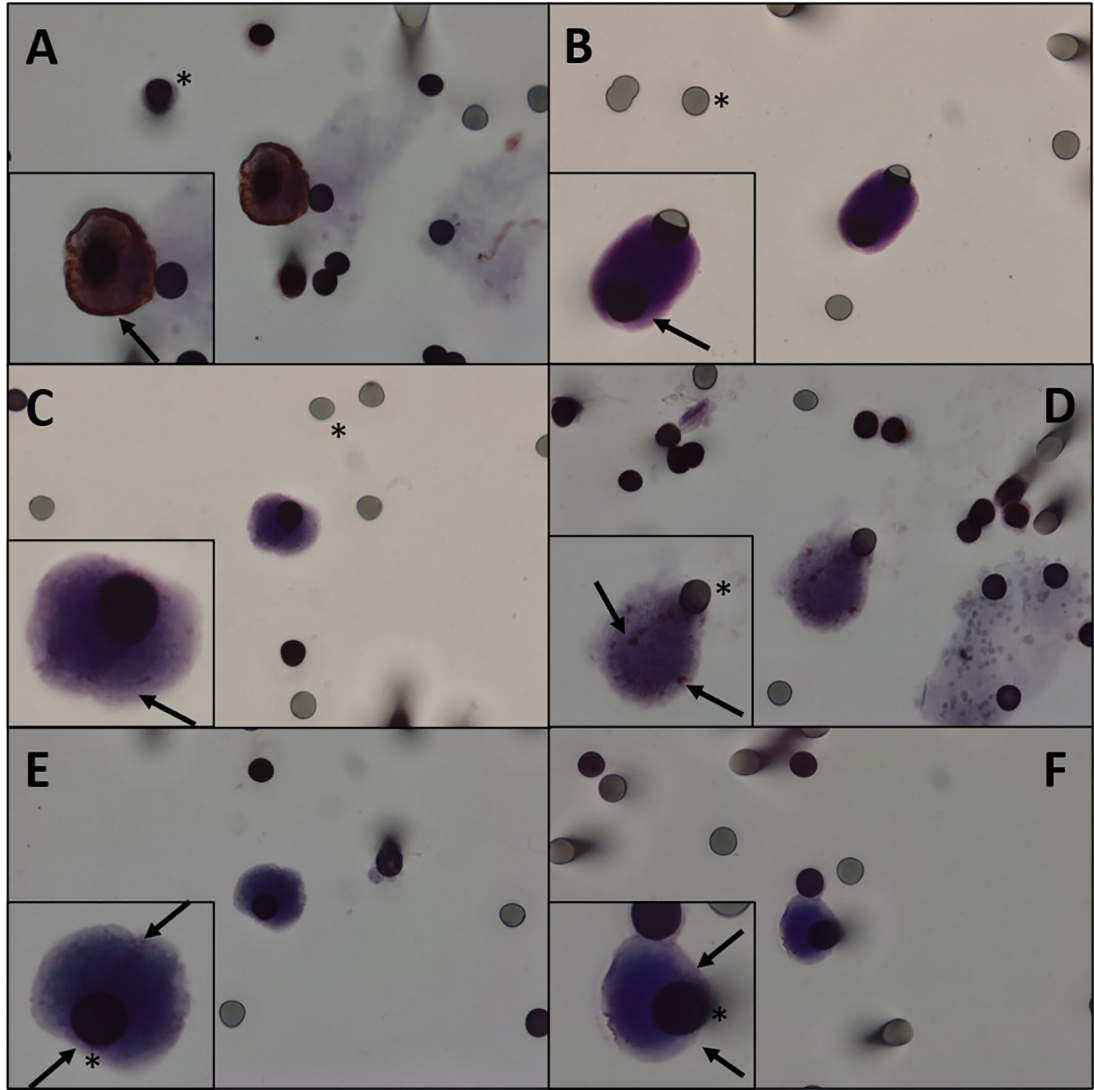
**(A)** Immunocytochemistry of CTC with β-catenin antibody counterstaining with DAB. **(B–F)** Immunocytochemistry of CTC with β-catenin antibody and counterstaining with magenta chromogen. We can observe different degrees of staining. **(A, B)** β-Catenin highly positive staining in a patient. **(C, D)** β-Catenin middle staining in a patient. **(E, F)** β-Catenin weakly positive staining in a patient. Black asterisk: membrane spots. Black arrows: β-catenin. CTC, circulating tumor cell.

**Figure 3:**
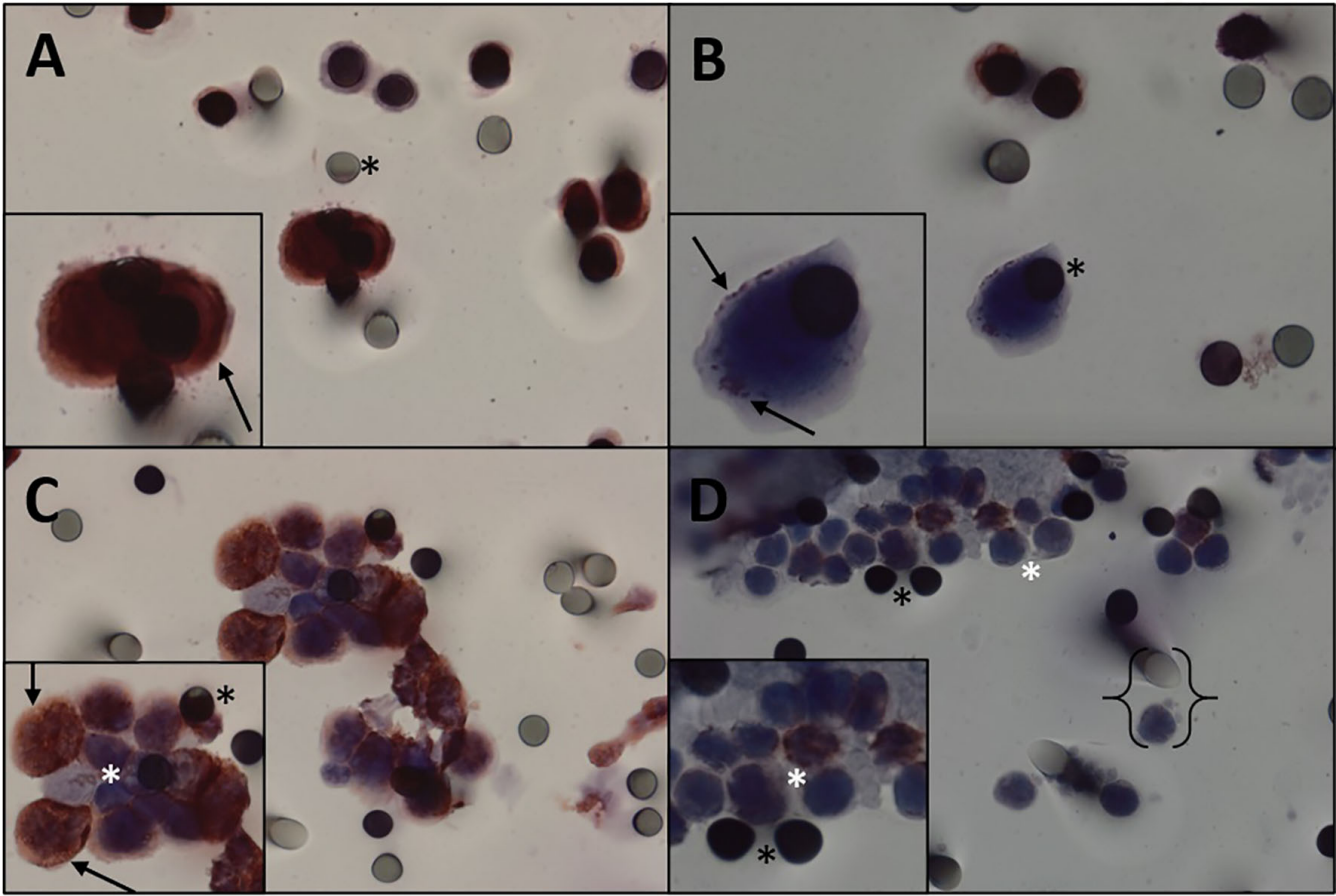
Immunocytochemistry for vimentin. **(A, B)** Immunocytochemistry of CTC with vimentin antibody and counterstaining with DAB. **(A)** High staining. **(B)** Weak staining. **(C)** Desmoid tumor CTMs observed in the blood filtered using the ISET, stained with vimentin and counterstained with DAB chromogen. **(D)** Leukocytes from desmoid tumor patient. Black asterisk: membrane spots. Black arrows: vimentin expression. White asterisk: leukocytes. Keys: size comparison between a leukocyte and a spot. CTC, circulating tumor cell; CTMs, circulating tumor microemboli; ISET, isolation by size of epithelial tumor cells.

CTCs of 11 patients (61.1%) expressed β-catenin. Immunohistochemical analysis of primary tumors revealed that 11 patients were positive for β-catenin (**Figure 2**) and three were negative, and the results of four patients were not reported. Five patients expressed β-catenin in both CTCs and primary tumors, and one who tested negative in the primary tumor also tested negative in CTCs. Two patients were positive for β-catenin expression in CTCs and negative in primary tumors (**Table 2**). Because i) IHC is the gold standard method for β-catenin detection in primary tumors and ii) primary tumor β-catenin expression was available in the medical records of only 14 patients, we found a concordance rate between primary tumors and CTCs of 42.8% (6 concordant/14 samples). As concordance, we considered simultaneous positive or negative expression of a given protein in both CTCs and primary tumors. Among the 18 patients evaluated for protein expression in CTCs, vimentin was observed in five patients (27.7%) (**Figure 3**), while nine were positive for TGFβ-RI (50%) and seven for COX-2 (38.8%).

## Discussion

Although it was our first study with DT, we have extensive experience with ISET, as we have been working with this method for a long time, with many published papers (29, 32–40) including two with sarcomas (29, 41). In the first paper with sarcoma (29), we tested the sensitivity of the method, as we were the first group to test ISET for non-solid tumors, and we proved, with cell line, that its sensitivity was high, even for mesenchymal cells. After spiking 25, 50, 100, and 150 HT1080 cells (derived from human fibrosarcoma), in triplicate, in 1 ml of blood from healthy donors and filtering the blood using ISET, we found mean numbers of 25, 54, 100, and 155 cells, respectively. The numbers found were not identical to the numbers of cells spiked because tumor cells were counted in a counting chamber, by dilution, and not one by one. In this study, we also filtered blood samples from 30 healthy donors in ISET and found no CTCs.

Since DTs do not metastasize, it is apparently counterintuitive to find CTCs in the bloodstream of these patients. However, our study showed that CTCs can be detected and isolated from DT patients even from relatively low volumes of blood samples. This finding suggests that CTCs might be playing a different role on the DT biology. That allowed us to hypothesize that CTCs can be working as vehicles that reseed the primary tumor, which could potentiate its aggressiveness. CTCs are characterized by cytomorphological features that include nucleus size ≥12 µm; hyperchromatic and irregular nuclei; visible presence of cytoplasm; and a high nucleus–cytoplasm ratio (31). We defined CTCs as cells displaying all the aforementioned characteristics, and we also observed CTC pleomorphism (**Figure 1** and **Supplementary Figure 2**). Morphologic changes resulting in more rounded or “fibroblastoid” CTCs were most likely acquired during *in vitro* cell handling or by the filtering process. In **Supplementary Figure 3**, examples of macrophages isolated from the blood of patients with DT can be appreciated, demonstrating the morphological differences between CTCs and macrophages.

The high prevalence of CTCs in our patient population might be explained by the self-seeding hypothesis. It has been proposed that self-seeding can accelerate tumor growth, angiogenesis, and stromal recruitment through seed-derived factors (27). Therefore, tumor self-seeding might explain the relationships among anaplasia, tumor size, vascularity, and prognosis and also suggests that local recurrences may result from seeding of disseminated cells following complete tumor excision (1, 42, 43).

CTMs were isolated from a single patient (**Figure 3** and **Table 2**) and were positive for β-catenin and vimentin expressions. Interestingly, it was in a 57-year-old woman whose diagnosis was in 2013 with the blood collection in this study made in September 2019 and who has not yet progressed. She has received only one treatment line (tamoxifen + celecoxib). Knowledge regarding CTMs, also called CTC clusters, has evolved over the last decade, and increasing evidence suggests that they play an essential role on the pathophysiology of metastasis (44). Preclinical and clinical studies exploiting other cancers have demonstrated that CTMs are associated with increased metastatic potential and poor prognosis (36, 45–48). Hayashi et al. detected CTMs in sarcoma patients using CellSieve™, a low-pressure microfiltration device. Our group previously isolated CTMs in sarcoma patients using ISET^®^ but did not address DTs (41). Taking this into account, together with our current data, it is reasonable to hypothesize that the inability of DTs to metastasize can be explained in part by their failure to generate CTMs (as we rarely found CTMs in our cohort of 18 patients). However, this hypothesis needs to be interpreted cautiously, due to the small sample size of our cohort.

β-Catenin is broadly used for DT diagnosis. In line with this, we thought that this protein could also be expressed in CTCs. Analysis of β-catenin protein expression in CTCs showed 35.7% of concordance with primary tumors (considering both i) patients from whom reports of the primary tumor were available and ii) those patients in whom the spots analyzed for β-catenin did not present CTCs). β-Catenin is encoded by the *CTNNB1* gene in humans and is involved in cell signaling, acting primarily as a transcription factor. It has an essential role on developmental biology and cell adhesion. Mutations and overexpression of this gene are associated with several types of cancers, such as lung, breast, ovarian, endometrial, hepatocellular, and colorectal carcinomas (19–21, 49). We also identified vimentin expression in the CTCs of five patients (27.7%). Vimentin is required for plasticity of mesenchymal cells under normal physiological conditions and migration of cancer cells that have undergone epithelial/mesenchymal transition. In a previous study of sarcoma CTCs conducted by our group, we detected vimentin in CTCs of three patients in a cohort of 11 (29).

TGF-β is a secreted cytokine that regulates cell migration, differentiation, and proliferation (50). Depending on its expression level, TGF‐β has both proangiogenic and antiangiogenic properties. Angiogenic factors (vascular endothelial growth factor and basic fibroblast growth factor) increase TGF‐β expression when its levels are low. At high levels, TGF-β rebuilds basement membrane and inhibits the growth of endothelial cells and smooth muscle cells (51, 52). In this study, curiously, nine patients (50%) were positive for TGFβ-RI expression in CTCs. In another study by our group (37), TGF-βRI expression was associated with poor prognosis of locally advanced head and neck cancer. The identification of TGF-βRI in CTCs opens paths for the comprehensive exploitation of this pathway in DT.

COX enzymes play important roles on human physiology and various pathological conditions (53, 54). There is evidence for the critical involvement of COX-2 in many pathologies, including cancer (55, 56). Here, we also found a high frequency of DT patients expressing COX-2 in CTCs. Considering that DTs do not form metastases but show aggressive local invasion, it is possible that COX-2 plays an essential role on this process, which needs to be further investigated.

In general, we found that β-catenin was highly expressed in CTCs. Except for patient #6, we also found that at least one marker was expressed by CTCs across the whole cohort analyzed in this study, suggesting that evolution of protein expression in CTCs might be used as a biomarker that allows the non-invasive diagnosis of DT patients.

In this study, we showed that CTCs are composed of highly heterogeneous cell populations with very different phenotypes. This difference in phenotypes makes the identification of CTCs challenging, due to their similarities with other cells of the immune system, such as giant monocytes and micromegakaryocytes. However, training in analysis with the help of cytopathological criteria can overcome these difficulties. Laget et al. (30) described results that consistently show the feasibility of isolating live and fixed tumor cells with a lower limit of detection (LLOD) of a cancer cell per 10 ml of blood and an LLOD sensitivity ranging from 83% to 100%. Those results demonstrated that ISET^®^ allows highly sensitive and impartial isolation of fixed tumor cells from blood for reliable identification of CTCs, as well as the development of immuno-molecular studies. Here, we were able to demonstrate the expression of mesenchymal proteins in CTCs by ICC, which are probably involved in tumorigenesis process.

To conclude, our study opens the prospect of using CTCs to predict desmoid dynamics throughout the course of the disease. In addition, it demonstrates that DTs release CTCs, opening new avenues for studying the biology of this tumor and improving our understanding of its high local relapse rates without distant metastasis. Additional studies with larger sample sizes should be conducted to validate our findings and explore the mechanism of DT development and progression.

## Data Availability Statement

The datasets presented in this study can be found in online repositories. The names of the repository/repositories and accession number(s) can be found in the article/**Supplementary Material**.

## Ethics Statement

The studies involving human participants were reviewed and approved by AC Camargo Cancer Center. The patients/participants provided their written informed consent to participate in this study.

## Author Contributions

AB: design, data analysis and interpretation, manuscript writing and collection and/or assembly of data. FC, MS and FP: clinical survey/review and writing. EA and AR: collection and/or assembly of data. TM: English review. CM: conception/design and writing of the clinical part of the manuscript. LC: conception/design, data analysis and interpretation, manuscript writing, final approval of manuscript. All authors contributed to the article and approved the submitted version.

## Funding

We thank the National Institute for Science and Tecnology in Oncogenomics and Therapeutic Innovation (INCT) for financial support for this study. ACB had a PhD fellowship from São Paulo Research Foundation FAPESP (2019/18100-8).

## Conflict of Interest

The authors declare that the research was conducted in the absence of any commercial or financial relationships that could be construed as a potential conflict of interest.

## Publisher’s Note

All claims expressed in this article are solely those of the authors and do not necessarily represent those of their affiliated organizations, or those of the publisher, the editors and the reviewers. Any product that may be evaluated in this article, or claim that may be made by its manufacturer, is not guaranteed or endorsed by the publisher.

## Supplementary Material

Supplementary Figure 1 **(A, B)** Positive control, A549 cell line “spiked” in healthy blood and stained for β-catenin and TGF β-RI respectively. **(C, D)** Positive control, U-87 cell line “spiked” in healthy blood and stained for COX-2 and Vimentin respectively. **(E)** Negative control, A-549 cell line “spiked” in healthy blood and negative for β-catenin. Cells were analyzed by light microscopy (BX61-Olympus).

Supplementary Figure 2 **(A–I)** Haematoxylin stained cells. Examples of isolated desmoid tumor CTCs with characteristic cytomorphological features (nucleus size ≥ 12 µm, hyperchromatic and irregular nucleus, visible presence of cytoplasm, and a high nucleus–cytoplasm ratio (31). White asterisk: leukocytes. Black asterisks: ISET membrane pore.

Supplementary Figure 3 **(A)** Leukocytes from desmoid tumor patient. **(B, C)** Examples of macrophages isolated from the blood of patients with desmoid tumor. In **(B)**, a macrophage stained with COX-2. In **(C)**, a macrophage visualized with haematoxylin.
