# Supplementary figures and images for: Circulating Tumor Cells in Desmoid Tumors: New Perspectives
Source: Front Oncol. 2021 Sep 14;11:622626. doi: 10.3389/fonc.2021.622626 (PMC8476862; doi:10.3389/fonc.2021.622626)

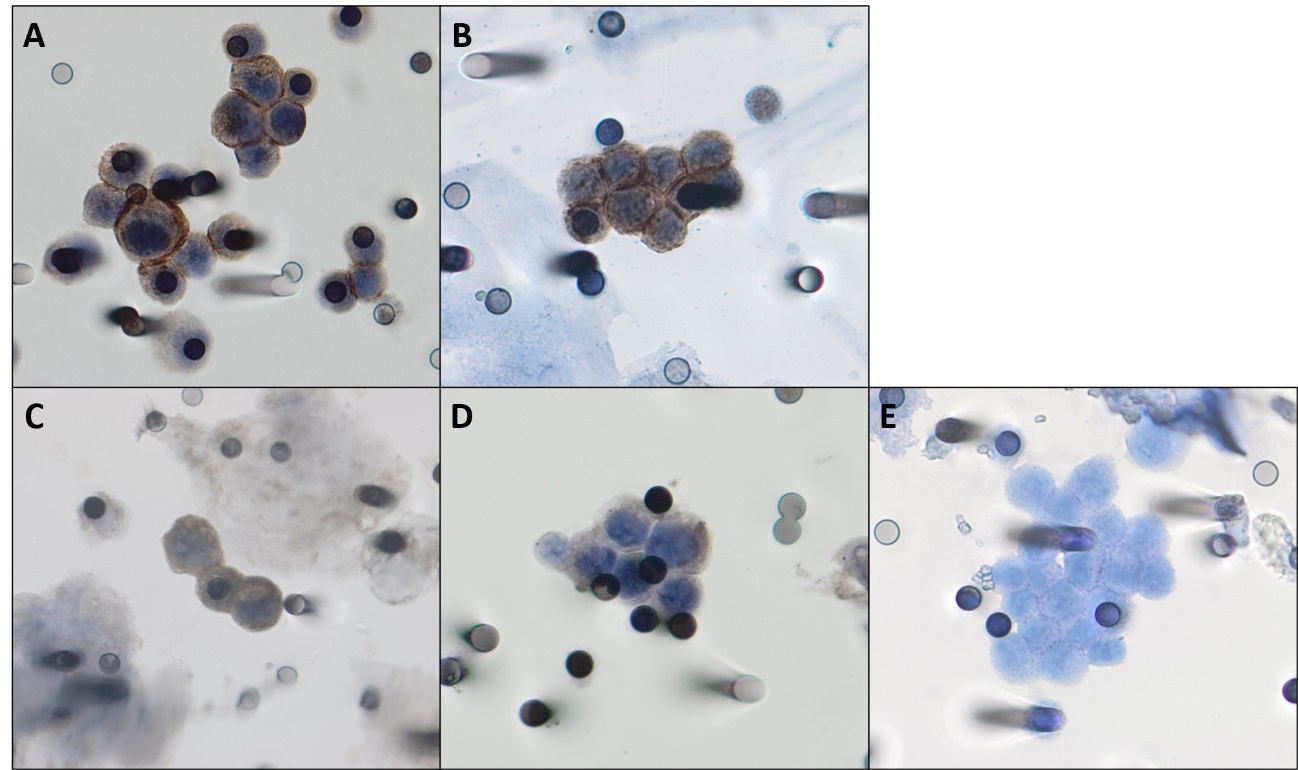

Supplement: Supplementary Figure 1 — (A, B) Positive control, A549 cell line “spiked” in healthy blood and stained for β-catenin and TGF β-RI respectively. (C, D) Positive control, U-87 cell line “spiked” in healthy blood and stained for COX-2 and Vimentin respectively. (E) Negative control, A-549 cell line “spiked” in healthy blood and negative for β-catenin. Cells were analyzed by light microscopy (BX61-Olympus). [file Image_1.jpeg]

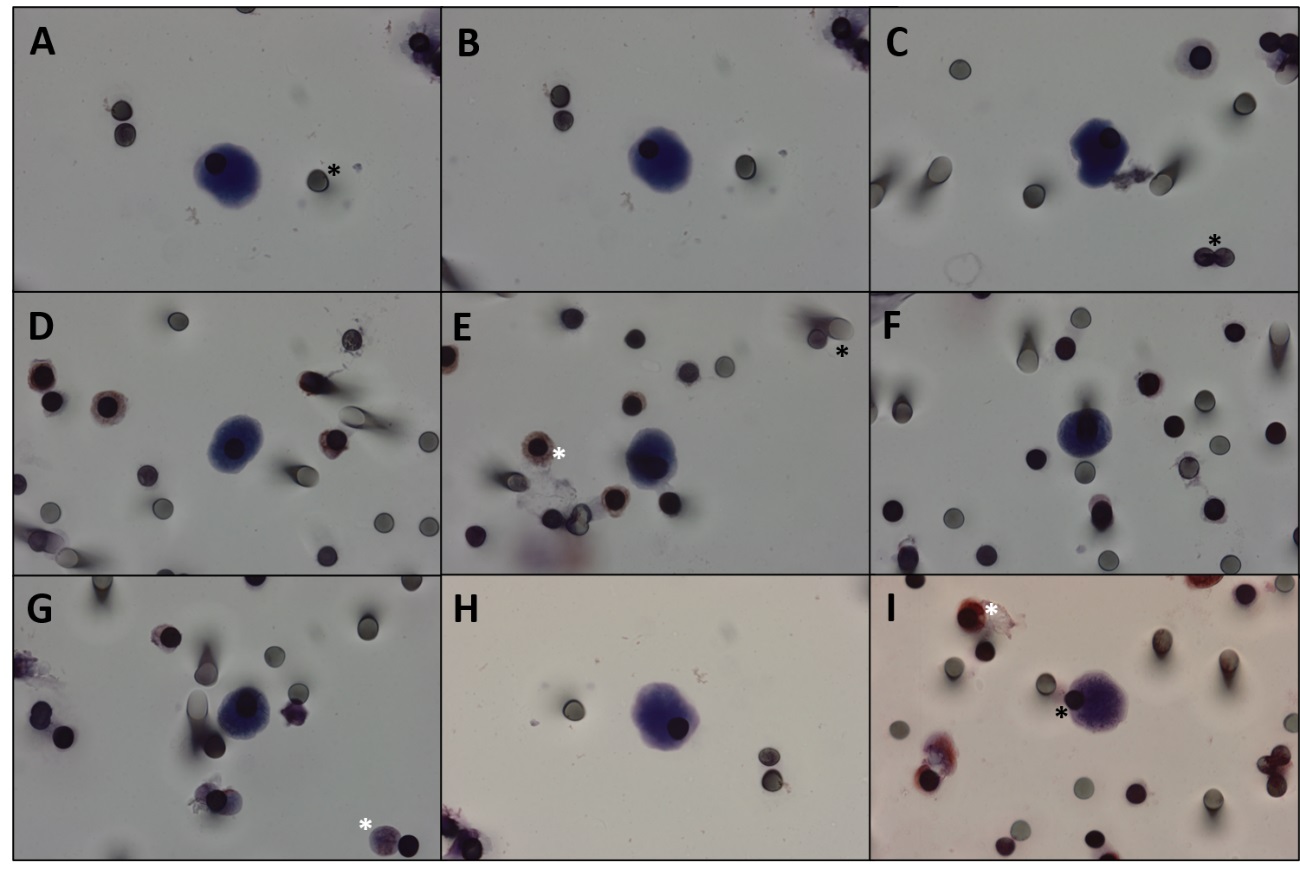

Supplement: Supplementary Figure 2 — (A–I) Haematoxylin stained cells. Examples of isolated desmoid tumor CTCs with characteristic cytomorphological features (nucleus size ≥ 12 µm, hyperchromatic and irregular nucleus, visible presence of cytoplasm, and a high nucleus–cytoplasm ratio (31). White asterisk: leukocytes. Black asterisks: ISET membrane pore. [file Image_2.jpeg]

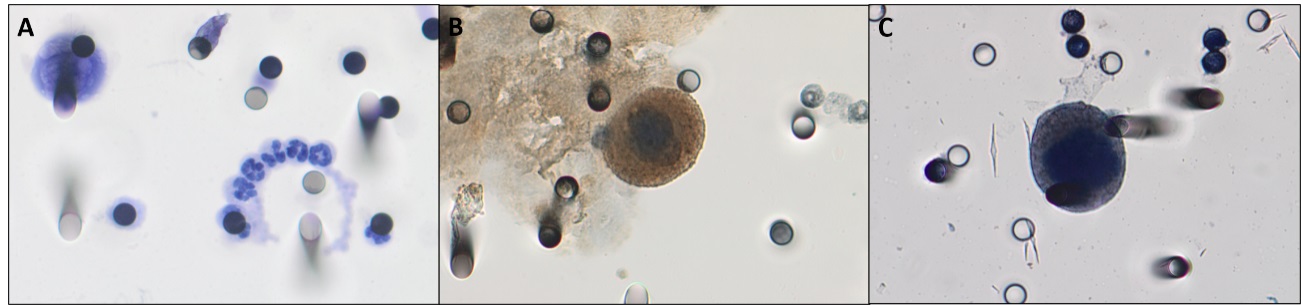

Supplement: Supplementary Figure 3 — (A) Leukocytes from desmoid tumor patient. (B, C) Examples of macrophages isolated from the blood of patients with desmoid tumor. In (B), a macrophage stained with COX-2. In (C), a macrophage visualized with haematoxylin. [file Image_3.jpeg]
